# A prospective randomised cross-over study of the effect of insulin analogues and human insulin on the frequency of severe hypoglycaemia in patients with type 1 diabetes and recurrent hypoglycaemia (the HypoAna trial): study rationale and design

**DOI:** 10.1186/1472-6823-12-10

**Published:** 2012-06-22

**Authors:** Peter Lommer Kristensen, Ulrik Pedersen-Bjergaard, Henning Beck-Nielsen, Kirsten Nørgaard, Hans Perrild, Jens Sandahl Christiansen, Tonny Jensen, Hans-Henrik Parving, Birger Thorsteinsson, Lise Tarnow

**Affiliations:** 1 Department of Cardiology, Nephrology and Endocrinology, Hillerød University Hospital, Dyrehavevej 29, DK-3400, Hillerød, Denmark; 2 Department of Endocrinology M, Odense University Hospital, Odense, Denmark; 3 Department of Endocrinology, Hvidovre University Hospital, Hvidovre, Denmark; 4 Department of Internal Medicine, Bispebjerg University Hospital, Copenhagen, Denmark; 5 Department of Endocrinology M, Aarhus University Hospital, Aarhus, Denmark; 6 Department of Medical Endocrinology, Copenhagen University Hospital (Rigshospitalet), Copenhagen, Denmark; 7 Steno Diabetes Center, Gentofte, Denmark; 8 Faculty of Health Sciences, University of Southern Denmark, Odense, Denmark; 9 Faculty of Health Sciences, University of Aarhus, Aarhus, Denmark; 10 Faculty of Health Sciences, University of Copenhagen, Copenhagen, Denmark

**Keywords:** Type 1 diabetes, Severe hypoglycaemia, Human insulin, Insulin analogues, PROBE

## Abstract

**Background:**

Severe hypoglycaemia still represents a significant problem in insulin-treated diabetes. Most patients do not experience severe hypoglycaemia often. However, 20% of patients with type 1 diabetes experience recurrent severe hypoglycaemia corresponding to at least two episodes per year. The effect of insulin analogues on glycaemic control has been documented in large trials, while their effect on the frequency of severe hypoglycaemia is less clear, especially in patients with recurrent severe hypoglycaemia. The HypoAna Trial is designed to investigate whether short-acting and long-acting insulin analogues in comparison with human insulin are superior in reducing the occurrence of severe hypoglycaemic episodes in patients with recurrent hypoglycaemia. This paper reports the study design of the HypoAna Trial.

**Methods/design:**

The study is a Danish two-year investigator-initiated, prospective, randomised, open, blinded endpoint (PROBE), multicentre, cross-over trial investigating the effect of insulin analogues versus human insulin on the frequency of severe hypoglycaemia in subjects with type 1 diabetes. Patients are randomised to treatment with basal-bolus therapy with insulin detemir / insulin aspart or human NPH insulin / human regular insulin in random order. The major inclusion criterion is history of two or more episodes of severe hypoglycaemia in the preceding year.

**Discussion:**

In contrast to almost all other studies in this field the HypoAna Trial includes only patients with major problems with hypoglycaemia. The HypoAna Trial will elucidate whether basal-bolus regimen with short-acting and long-acting insulin analogues in comparison with human insulin are superior in reducing occurrence of severe hypoglycaemic episodes in hypoglycaemia prone patients with type 1 diabetes. http://www.clinicaltrials.gov: NCT00346996.

## Background

Severe hypoglycaemia still represents a significant risk for many patients with type 1 diabetes. The condition is feared by patients [1,2] and relatives [3] and remains a major barrier in achieving recommended glycaemic targets [4]. Moreover, severe hypoglycaemia may, although rarely, lead to permanent brain damage or death [5]. The frequency of severe hypoglycaemia in type 1 diabetic populations is 1–1.7 episodes per patient-year [6-14]. The distribution of episodes is, however, very skewed. Thus, 20% of patients with type 1 diabetes experience two or more episodes of severe hypoglycaemia per year [15]. Once prone to recurrent severe hypoglycaemia, patients often remain so throughout their life.

Insulin analogues were developed to improve treatment of insulin-treated diabetes with respect to glycaemic control and avoidance of hypoglycaemic episodes. Short-acting insulin analogues (aspart, lispro and glulisine) were designed to mimic the fast physiological postprandial insulin release and long-acting insulin analogues (detemir and glargine) were designed to mimic the basal continuous insulin release with minimal peak action, thereby leading to a presumed decreased risk of hypoglycaemia. The effect of insulin analogues on glycaemic control has been documented in large trials [16,17]. However, for several reasons the impact of insulin analogues on the frequency of severe hypoglycaemia is less clear. Firstly, most studies comparing the effects of human insulin with those of insulin analogues specifically exclude patients at risk of severe hypoglycaemia, such as patients with previous severe events or impaired hypoglycaemia awareness, from participation [18-21]. This renders the trials insufficiently statistically powered to detect differences in the frequency of severe hypoglycaemia and the conclusions from these studies can not readily be extrapolated to patient groups prone to severe hypoglycaemia. Secondly, hypoglycaemia is often less well defined and detected, at best as a secondary endpoint, with reports including a mixture of mild, severe, and asymptomatic hypoglycaemia. Thirdly, severe hypoglycaemia should be analysed according to a statistic model that takes into account the much skewed distribution of episodes e.g. negative binomial or zero-inflated models [22].

A Cochrane review comparing short-acting insulin analogues with human insulin included 49 studies, of which 10 studies were eligible in relation to hypoglycaemia [16]. The analysis showed that the overall mean occurrence of hypoglycaemic episodes was lower for short-acting analogues in comparison to regular insulin in patients with type 1 diabetes (−0.2 per patient per month (95% confidence interval: -1.1 to 0.7)). No statistical comparative calculations were possible specifically addressing severe hypoglycaemia due to inconsistent and bias-prone definitions in the different studies. However, in the analogue group the incidence of severe hypoglycaemia ranged from 0–247 (median 22) episodes per 100 patient-year compared to the incidence in the human insulin group ranging from 0–544 (median 46).

After the initiation of the present study, a meta-analysis based on 20 studies (duration > 12 weeks) was published comparing long-acting insulin analogues with long-acting human insulin in type 1 diabetes, including 12, 15 and 13 studies analysing any hypoglycaemia, severe hypoglycaemia, and nocturnal hypoglycaemia, respectively [17]. The analysis showed that long-acting analogues are not associated with a significant reduction of overall risk of any hypoglycaemia in comparison with NPH insulin. When episodes of severe hypoglycaemia and nocturnal hypoglycaemia were separately analysed, insulin detemir, but not insulin glargine, was associated with a reduced risk compared to human NPH insulin (OR 0.73 (0.60 - 0.89), p = 0.002 and OR 0.69 (0.55 - 0.86), p = 0.001; respectively). There are important limitations with the meta-analysis. Firstly, the trials use different criteria for hypoglycaemia, different methods in recording severe hypoglycaemia, and many trials are not randomised. Secondly, the participants in the studies are relatively young (mean: 34 years) with a short duration of diabetes (mean: 13 years) suggesting that the participants’ state of hypoglycaemia awareness is relatively unaffected. Thirdly, in some trials the number of insulin injections per day are different in the human insulin group and the insulin analogue group. Finally, the vast majority of the trials in the analysis were sponsored by manufacturers of long-acting insulin analogues. To our knowledge a direct comparison of long acting insulin analogues and long-acting human insulin has not been made in patients at high risk of severe hypoglycaemia.

When it comes to patients at high risk of severe hypoglycaemia, who may have the greatest potential benefit from insulin analogue therapy, there is only one small, randomised,12-month cross-over study comparing the effect of the short-acting insulin analogue (lispro) and regular human insulin on the frequency of severe hypoglycaemia [23]. The study population comprised 33 patients with type 1 diabetes and hypoglycaemia unawareness. There was a trend towards a lower incidence of severe hypoglycaemia during treatment with insulin lispro in comparison with human insulin (p = 0.087). Another study in 322 women with type 1 diabetes, who were pregnant or planning pregnancy and therefore being at relatively high risk of severe hypoglycaemia due to very tight glycaemic control, compared insulin aspart with human insulin as meal-time insulin (with human NPH insulin as basal insulin) using an open-label, randomised, parallel-group, multicentre design. Although the risk of severe hypoglycaemia in women treated with insulin aspart was 28% lower, the difference did not reach statistical significance [24]. In a randomised 24-week pilot study of 15 patients with type 1 diabetes complicated by severe hypoglycaemia, comparing rigorous hypoglycaemia avoidance with insulin analogue therapy (lispro and glargine), continuous subcutaneous insulin therapy (CSII) or education alone (but not including any control group), hypoglycaemia awareness was restored and further severe hypoglycaemia was prevented with concomitant improvement in glycaemic control in the analogue and CSII groups [25].

The HypoAna Trial is designed to elucidate whether short-acting and long-acting insulin analogues (insulin aspart and insulin detemir) in comparison with human regular and NPH insulin are superior with respect to reducing the occurrence of severe hypoglycaemic episodes in patients with recurrent hypoglycaemia. There is an urgent need for this evidence in clinical practice and a need to elucidate if the higher costs of insulin analogues are justified in this respect. However, since the structures of different insulin analogues are not similar, the results of this study cannot be directly extrapolated to other insulin analogues (lispro, glulisine and glargine).

## Objective

The primary objective is to evaluate the effect of treatment with insulin analogue and human insulin over the preceding 9 months in each treatment arm on the incidence of severe hypoglycaemia in patients with type 1 diabetes prone to hypoglycaemia.

The secondary objectives are – with reference to the ADA criteria for classification of hypoglycaemia [26] – to evaluate the effects of insulin analogue and human insulin from 3 to 12 months of treatment on the incidence of:

· documented (mild) symptomatic hypoglycaemia.

· asymptomatic hypoglycaemia.

· severe hypoglycaemia, documented symptomatic hypoglycaemia, and asymptomatic hypoglycaemia during daytime.

· severe hypoglycaemia, documented symptomatic hypoglycaemia, and asymptomatic hypoglycaemia during night-time

## Methods/design

### Design

**Figure 1:**
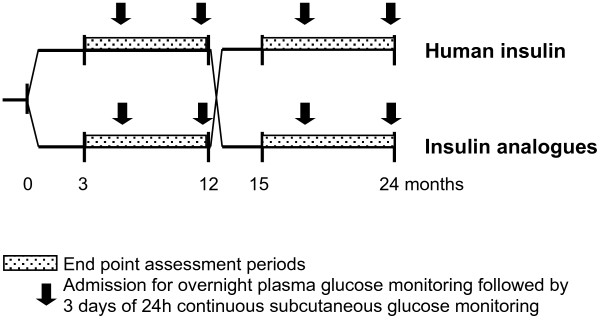
Graphic presentation of the HypoAna study which is a prospective randomised cross-over study of the effect of insulin analogues and human insulin on the frequency of severe hypoglycaemia in patients with type 1 diabetes and recurrent hypoglycaemia.

The study is a Danish two-year, investigator-initiated, prospective, randomised, open, blinded endpoint (PROBE), multicentre, cross-over trial investigating the effect of insulin analogues versus human insulin on the frequency of severe hypoglycaemia (Figure 1). Patients are randomised to treatment with basal-bolus therapy with insulin detemir and insulin aspart (Levemir®/Novorapid®) or human NPH insulin and human regular insulin (Insulatard®/Actrapid®), in random order. Doses of insulin are adjusted according to individual patient need at the discretion of the investigator. Thus, a treat-to-target design is deliberately not used in order not to intervene with present glycaemic control and everyday clinical practice, which otherwise in itself will interfere with the risk of hypoglycaemia. The best obtainable glycaemic control for the individual patient is strived for in both treatment periods. The protocol did not specify the timing and number of insulin injections per day. In general, a four times daily basal-bolus regimen was selected, e.g. basal insulin before bedtime and prandial insulin before meals. Patients were instructed in insulin injection technique at the beginning of each treatment arm. Endpoints are assessed during the last 9 months of each treatment arm, the first 3 months being used for run-in on a new treatment regimen. Optional admissions for overnight plasma glucose measurements followed by three days of blinded continuous glucose monitoring (CGM) are performed after 6 and 12 months of treatment in each treatment arm.

### Subjects

From November 2006 to June 2007 questionnaires were sent to 6112 patients with type 1 diabetes attending the outpatient clinics at Steno Diabetes Center, Hillerød Hospital, Hvidovre Hospital, Bispebjerg Hospital, Odense University Hospital, Copenhagen University Hospital (Rigshospitalet), and Aarhus University Hospital. From some centres reminders were sent once. A total of 3861 patients filled in the questionnaire (63.2%). Patients were, among other things, asked how many episodes of severe hypoglycaemia they had experienced during the last year. Patients with two or more episodes were identified (n = 720) and invited by letter to participate in the HypoAna Trial. Subjects were included in the HypoAna Trial from May 2007 to November 2009. The inclusion criteria are type 1 diabetes for more than five years, two or more episodes of severe hypoglycaemia in the previous year (defined as need for third party assistance to restore blood glucose level), age > 18 years, and a negative pregnancy test. There were no inclusion criteria regarding the pre-randomisation insulin treatment. Exclusion criteria are treated adrenal or growth hormone insufficiency or untreated hypothyroidism, myocardial infarction or coronary revascularisation, transient ischemic attack or stroke within the last three months, history of malignancy unless a disease-free period exceeding five years, history of alcohol or drug abuse, pregnancy or lactation, and women of childbearing potential who are not using chemical or mechanical contraception. Criteria for discontinuation are voluntary withdrawal of consent, pregnancy, or non-compliance with the study protocol as judged by the investigator. Discontinuing patients are not substituted, but data on discontinuing patients are included in data analyses on an intention-to-treat basis. The study was approved by The Regional Committee on Biomedical Research Ethics (# H-KA-20070008) and the Danish Medicines Agency (# 2612–3397). The study is registered at http://www.clinicaltrials.gov (# NCT00346996). The Study will be conducted in accordance with the Helsinki Declaration.

### Experimental protocol

Participants attend the outpatient clinics every three months. After informed consent the participants meet fasting at the first visit in the outpatient clinic at 08:00 a.m. Information about demography, lifestyle and clinical characteristics are gathered. Weight and height are measured without shoes. Blood pressure is measured twice after 10 minutes rest in a sitting position. As a measure of peripheral and autonomic neuropathy, biothesiometry, beat-to-beat variation, and orthostatic blood pressure responses are assessed. A 12-lead electrocardiogram is recorded and visually assessed. State of hypoglycaemia awareness is classified by a validated method [27,28]. Patients are asked: “Do you have symptoms when you have a hypo?” The subjects can answer “always” (normal awareness), “usually” (impaired awareness), and “occasionally” or “never” (unawareness). The frequencies of episodes of mild hypoglycaemia per week (defined as manageable by the patient) and severe hypoglycaemia in the previous year are determined on the basis of the aforementioned questionnaires used for screening of patients for the present trial. Recall of severe hypoglycaemia is well preserved during a one-year period [27]. Fasting blood samples are taken and samples for later analyses are centrifuged, processed and stored immediately at −80°C. HbA1c and C-peptide concentrations are measured centrally at Steno Diabetes Center. At the end of visit 1 a 30-min ACTH test (Synacthen®, 0.25 mg i.v.) is performed. After a breakfast in the outpatient clinic the patient’s insulin injection technique is tested and corrected if needed. Three questionnaires about quality of life are filled in. The EuroQol-5 dimension quality of life index (EQ-5D), the Insulin Treatment Satisfaction Questionnaire (ITSQ) and the Hypoglycemia Fear Survey (HFS). EQ-5D is a commonly used, short questionnaire assessing health-related quality of life. Patients describe their health status on five domains (mobility, self-care, usual activities, pain/discomfort, anxiety/depression) with three different answers: “I have no problem”, “some problem”, or “extreme problem”. Furthermore, patients rate health state on a visual analogue scale [29,30]. The ITSQ measures treatment satisfaction concerning the insulin regimen and comprises 22 questions about different aspects of insulin treatment [31]. The HFS comprises 33 questions and measures to what degree patients change daily practise to avoid hypoglycaemia and measures different aspects of worries of episodes of hypoglycaemia [32]. At the end of the visit patients are centrally randomised at Steno Diabetes Center by an assistant not further involved in the trial, based on site-specific computer-generated randomisation lists in blocks of four patients. Finally, free study medication is handed out.

### Endpoint registration

#### Severe hypoglycaemia

In case of a suspected episode of severe hypoglycaemia, patients are instructed to make a telephone call within 24 hours to a call center served by a trained diabetes nurse. The nurse performs a structured interview with the patient to validate the severity (need for assistance from others) and the plausibility of the event according to Whipple’s triad: I) characteristic hypoglycaemic symptoms; II) biochemical confirmation with plasma glucose ≤ 3.9 mmol/l; and III) adequate response to treatment with carbohydrates, glucose or glucagon. According to Whipple’s triad only episodes fulfilling all criteria can be considered definite, whereas those fulfilling two criteria can only be considered probable. Questions about time, place, blood glucose, possible cause(s) of hypoglycaemia, and types of help given to the patient are asked along with questions about health-related quality of life and questions about any consequences of the episode regarding physical or material damage and work-related absenteeism. Furthermore, at every visit to the outpatient clinic patients are asked if they have experienced any episodes of severe hypoglycaemia not recorded by telephone interview. In such cases an interview is carried out by the patient’s doctor or nurse using the same questionnaire as mentioned above. All telephone interviews are done by the same two study nurses (blinded to the treatment modality) located at Hillerød Hospital. Data from the last 9 months of each treatment arm will be used for endpoint analyses.

#### Mild symptomatic and asymptomatic hypoglycaemia

Patients are instructed and strongly encouraged to make a 7-point self-monitored blood glucose (SMBG) profile two days a week in the entire study period. All values are entered into a diary with study-specific guidance and at every outpatient visit the diary is evaluated by the investigator and copies of the relevant pages are saved as source data. If patients measure a SMBG value ≤ 3.9 mmol/l, they are instructed to write down if they have concomitant symptoms of hypoglycaemia. All SMBG values are measured with the Ascensia® CONTOUR® blood glucose monitoring system (Bayer Health Care, Leverkusen, Germany).

#### Nocturnal hypoglycaemia

Patients are instructed to measure a nocturnal blood glucose once every month at 3 a.m. Moreover, patients are invited (optional) to stay overnight at Steno Diabetes Center four times during the study (two times at month 6 and 12 in every treatment arm). In the evening before bedtime, a study-specific blinded continuous glucose monitoring (CGM) device (Guardian® REAL-time (with a “black” display), Medtronic Minimed, Northridge, USA) is mounted by a trained study nurse and the patient is instructed how to calibrate the device, using capillary glucose measurements. Thereafter, a venous line is inserted in an anticubital vein. During the night, blinded samples for subsequent plasma glucose measurements are drawn once every hour, while the patient is sleeping.

#### CGM

Interstitial glucose concentrations are assessed for three days in patients who accept the four optional overnight stays at Steno Diabetes Center, i.e. 12 days of CGM in total. After the three days the glucose sensor and monitor are collected and data are downloaded to a computer for further analysis. These data remain blinded until the end of the study and are not used to control glycaemic levels during the study period. Special blinded versions of the CGM device with a “black screen” were supplied by the manufacturer. The CGM data will provide a unique possibility to assess the accuracy of CGM and compare time spent at hypoglycaemia, number of unrecognized episodes of hypoglycemia in this selected patient population.

#### HbA1c and plasma glucose

At every visit to the outpatient clinic (totalling 9 visits per patient) blood samples are drawn to measure HbA1c levels (analysed centrally at Steno Diabetes Center) and random plasma glucose concentrations (measured locally).

### Power calculation

In the power calculation, the number of events in each treatment period is assumed to be distributed according to a Poisson model. No period effect exceeding the 3 months of run-in is assumed. Based on the total number of observed events for each individual patient, the number of events for a treatment period is reduced to a binomial distribution:

Binomial (total number of events, λ_human insulin_/(λ_human insulin_ + λ_insulin analogue_)), which under the null hypothesis of no difference in event rate between insulin analogues and human insulin becomes binomial (total number of events, ½). The power calculations are thus performed with the use of an exact test of number of events in a binomial distribution with the probability of p = 1/2 versus alternatively with the probability of P = λ_human insulin_/(λ_human insulin_ + λ_insulin analogue_) = 1/(1+ λ_insulin analogue_/λ_human insulin_). The sample size calculation thus indicates the total number of events needed. The study population is selected to be patients with an expected increased risk of severe hypoglycaemia. Assuming an incidence of 2.8 severe hypoglycaemic events per patient-year, corresponding to 2.09 per 9 months treatment period, and setting minimal relevant difference (MIREDIF) to a reduction in the incidence of severe hypoglycaemia of 15% (based on the conclusion of the American Diabetes Association Workgroup on Hypoglycemia ”that any significant reduction in severe hypoglycemia (that requiring the assistance of another individual), even by as little as 10–20%, would be advantageous” [26]), this will correspond to a reduction in the relation between human and analogue insulin of: λinsulin analogue/λhuman insulin = 0.85. Setting power to 80% and a test level of 5%, the total number of events needs to be 1220. With an expected number of events of 2.09 + 1.75 (1.75 = 2.09 x 15% reduction in incidence of severe hypoglycaemia) = 3.84 events per 18 months’ treatment, this corresponds to a need for 315 patients. With an expected dropout rate of 15%, 370 patients need to be included.

### Statistics

The crossover design makes it possible to compare the two treatment modalities within the same person, thereby minimising between-patient variation. This design is appropriate because type 1 diabetes is a chronic, stable disease with slow progression. Therefore, the status of the patient in the first treatment period is assumed to be the same in the second period. To avoid a possible period effect and unwelcome fluctuations in blood glucose in relation to shift from one treatment modality to the next, registration of the primary endpoint is performed after three months in each arm. The long participation of two years per patient ensures a sufficient time of observation to minimise the risk of dealing with patients at particularly good or bad periods of their illness. Therefore, the analysis will be based on data from patients who complete the first treatment period and at least 6 months of the second treatment period. An analysis in a negative binomial distribution or Poisson model will be used to describe the number of events through a log link function as a linear function of treatment, treatment period, HbA_1c_ levels and other relevant variables (state of hypoglycaemia awareness, C-peptide status, age, duration of diabetes, daily insulin dose). Individual differences in the observation period will be adjusted for in the analysis.

### Timeline

“Last patient, last visit” was November 2011.

### Laboratory analysis

At the first visit fasting C-peptide, haemoglobin, haematocrit, plasma creatinine, plasma sodium, and plasma potassium, thrombocyte count, white blood cell count, alanine aminotransferase, alkaline phosphatase, bilirubin, total cholesterol, high and low density lipoprotein cholesterols, triglycerides, thyroid-stimulating hormone TSH, T4 and T3 are measured. These analyses are repeated after 1 year (before changing insulin regimen) and after 2 years (final visit). Participants with C-peptide concentrations below 300 pmol/l or stimulated (venous blood glucose concentration >12 mmol/l) C-peptide concentrations below 600 pmol/l were considered C-peptide negative. At every visit HbA1c and random plasma glucose are measured. Biobank samples (serum, plasma and DNA) are gathered at baseline and six times (three times in each insulin regimen) during the study. Urinary sodium and albumin concentration are determined in urine samples collected from two separate nights at baseline and repeated after 1 year (before shifting insulin regimen) and after 2 years (final visit). HbA1c is measured centrally at Steno Diabetes Center using High Performance Liquid Chromatography method on a Tosoh Automated Glycohemoglobin Analyzer. C-peptide is measured with an AutoDELFIA C-peptide kit (detection limit 5 pmol/l). An ACTH stimulation test was undertaken at the first visit. All other analyses are performed locally by routine methods.

## Discussion

In contrast to almost all other studies in this field the HypoAna Trial includes only patients with major hypoglycaemic problems. The HypoAna Trial will elucidate whether short-acting and long-acting insulin analogues in comparison with human insulin are advantageous in reducing the number of hypoglycaemic episodes in these patients. If proven, this is of major importance to the patients and their health care professionals in planning optimal insulin regimens, and for the society from a cost-benefit point of view because costs are substantially higher for insulin analogues than for human insulin.

## Abbreviations

ACTH, Adrenocorticotropic Hormone; ADA, American Diabetes Association; CGM, Continuous glucose monitoring; EQ-5D, EuroQol-5 dimension quality of life index; HFS, Hypoglycemia Fear Survey; ITSQ, Insulin Treatment Satisfaction Questionnaire; MIREDIF, Minimal relevant difference; NPH, Neutral protamine Hagedorn; OR, Odds Ratio; PROBE, Prospective, randomised, open, blinded endpoint; SMBG, Self-monitored blood glucose; TSH, Thyroid-stimulating Hormone.

## Competing interests

HB-N, JSC and HP has received lecture fee from Novo Nordisk A/S. H-HP owns stock options in Novo Nordisk A/S. BT, KN and UP-B served on advisory boards for Novo Nordisk. LT holds shares in NovoNordisk A/S and is employed at Steno Diabetes Center, a public hospital owned by NovoNordisk A/S. The authors declare no other conflict of interest.

## Authors’ contributions

PLK, UP-B, HB-N, KN, HP, JSC, TJ, H-HP, BT and LT conceived the study, and participated in its design and coordination. PLK, UP-B, BT and LT drafted the manuscript. All authors have read and approved the final manuscript.

## Pre-publication history

The pre-publication history for this paper can be accessed here:

http://www.biomedcentral.com/1472-6823/12/10/prepub
